# Impact of early brain lesions on the optic radiations in children with cerebral palsy

**DOI:** 10.3389/fnins.2022.924938

**Published:** 2022-10-05

**Authors:** Rodrigo Araneda, Daniela Ebner-Karestinos, Laurance Dricot, Enimie Herman, Samar M. Hatem, Kathleen M. Friel, Andrew M. Gordon, Yannick Bleyenheuft

**Affiliations:** ^1^Institute of Neuroscience, Université Catholique de Louvain, Ottignies-Louvain-la-Neuve, Belgium; ^2^Exercise and Rehabilitation Science Institute, School of Physical Therapy, Faculty of Rehabilitation Science, Universidad Andrés Bello, Santiago, Chile; ^3^Physical and Rehabilitation Medicine, Brugmann University Hospital, Brussels, Belgium; ^4^Faculty of Medicine and Pharmacy, Vrije Universiteit Brussel, Brussels, Belgium; ^5^Faculty of Physical Education and Physiotherapy, Vrije Universiteit Brussel, Brussels, Belgium; ^6^Burke-Cornell Medical Research Institute, White Plains, NY, United States; ^7^Department of Biobehavioral Sciences, Teachers College, Columbia University, New York, NY, United States

**Keywords:** diffusion tensor imaging, early brain lesion type, hemiparesis, lesion side, white matter

## Abstract

Due to their early brain lesion, children with unilateral spastic cerebral palsy (USCP) present important changes in brain gray and white matter, often manifested by perturbed sensorimotor functions. We predicted that type and side of the lesion could influence the microstructure of white matter tracts. Using diffusion tensor imaging in 40 children with USCP, we investigated optic radiation (OR) characteristics: fractional anisotropy (FA), mean diffusivity (MD), axial diffusivity (AD) and radial diffusivity (RD). First, we compared the OR of the lesional and non-lesional hemisphere. Then we evaluated the impact of the brain lesion type (periventricular or cortico-subcortical) and side in the differences observed in the lesional and non-lesional OR. Additionally, we examined the relationship between OR characteristics and performance of a visuospatial attention task. We observed alterations in the OR of children with USCP on the lesional hemisphere compared with the non-lesional hemisphere in the FA, MD and RD. These differences were influenced by the type of lesion and by the side of the lesion. A correlation was also observed between FA, MD and RD and the visuospatial assessment mainly in children with periventricular and right lesions. Our results indicate an important role of the timing and side of the lesion in the resulting features of these children’s OR and probably in the compensation resulting from neuroplastic changes.

## Introduction

Cerebral palsy (CP) is a group of movement and posture disorders resulting from early brain injury. With an incidence of 4–10 per 1,000 children (Graham et al., 2016), CP is the most common cause of pediatric motor deficits, and is often accompanied by disabilities in cognitive and sensory functions (Krageloh-Mann and Cans, 2009; Weierink et al., 2013). These persistent deficits are attributable to brain structural abnormalities arising at different stages of brain development, with consequences for neuronal proliferation, migration, and differentiation, as well as neuronal growth and myelination (de Graaf-Peters and Hadders-Algra, 2006; Stiles and Jernigan, 2010). Therefore, the timing of the brain lesion influences the nature of subsequent perturbations in brain development (Chugani et al., 1996; Brizzolara et al., 2002). The two most common types of structural defect in children with unilateral spastic cerebral palsy (USCP) are periventricular lesions and cortical/subcortical lesions. Periventricular lesions arise early in the 3rd trimester of gestation and are associated mainly with white matter damage, whereas cortical/subcortical lesions, typically arise at the end of the 3rd trimester (Volpe, 1997; Cowan et al., 2003; Krageloh-Mann and Horber, 2007) and are associated mainly with gray matter damage (Bax et al., 2006; Krageloh-Mann and Horber, 2007; Reid et al., 2015). Periventricular lesions of the white matter mainly affect sensorimotor functions (Staudt et al., 2000, 2003; Pavlova and Krageloh-Mann, 2013). The cortical/subcortical lesions, on the other hand, also affect sensory and motor structures such as the basal ganglia and the primary motor cortex; the extent of a subcortical lesion is closely related to the severity of sensorimotor impairments (Martinez-Biarge et al., 2010). Less commonly, USCP may also arise from brain malformations occurring in the 2nd trimester of gestation (Volpe, 1997; Cowan et al., 2003; Krageloh-Mann and Horber, 2007).

Given that some functions such as language, visuospatial attention and (fine) hand motor control (Springer et al., 1999; Gotts et al., 2013) are lateralized, the side of the lesion may have some bearing on functional (re)organization of the developing brain. These lateralized functions accommodate differently depending on whether damage is congenital or acquired in the adult. For instance, a left brain lesion involving Broca’s or Wernicke’s areas will frequently induce permanent language impairments in adult stroke patients (Willmes and Poeck, 1993; Perani et al., 2003), whereas lesions in comparable regions of children with CP may result only in delayed language acquisition (Krageloh-Mann et al., 2017). Conversely, visuospatial abilities are usually lateralized to the non-dominant right hemisphere (Corballis, 2003). Adult stroke patients with right hemisphere lesions may show unilateral spatial neglect (Karnath et al., 2004), with absence of perception of the contralateral hemispace (Laplane and Degos, 1983). Interestingly, unilateral spatial neglect, notably in visual cancelation tasks (Laurent-Vannier et al., 2003; Trauner, 2003), is evident in children with early brain injury to either hemisphere (Thareja et al., 2012). This suggests spatial attention functions have greater plasticity in the damaged developing brain than in adults. Spatial neglect has been associated both with lesions in parietal cortex (Corbetta and Shulman, 2011) and in white mater tracts, including the optic radiations (OR) (Chechlacz et al., 2010). Although reorganization of motor pathways is well-documented in children with USCP (Cioni et al., 2011), less is known about corresponding reorganization of visuospatial functions. Guzzetta (2010) proposed a model wherein a primary visual cortex lesion would provoke reorganization of visual function in occipital areas extending beyond the primary visual cortex. On the other hand, subcortical lesions affecting the retro-geniculate pathway might entail remodeling of the OR to attain the preferred target in primary visual cortex. While intriguing, there has been little direct empirical evidence supporting this twofold model.

In the present study, we therefore aimed to (1) describe the reorganization of the OR in children with USCP, (2) investigate the effects of the type and side of the lesion on white matter changes in the OR of children with USCP due to an early brain lesion, and (3) assess the relationship between changes in the OR and performance of visuospatial attention tasks. We hypothesized that white matter characteristics of the OR would differ according to the side and type of lesion, and that OR findings would correlate with the extent of visuospatial neglect in children with USCP.

## Materials and methods

### Participants

Forty children with diagnosis of USCP participated in this study, of whom 26 were recruited at the Université Catholique de Louvain in Belgium, and 14 at Teachers College of Columbia University in New York City (19 girls; mean age: 9.1 ± 2.8 years; 22 right hemiparesis) from among children who participated or were interested in participating in intensive rehabilitation day camps (Bleyenheuft et al., 2020). Teachers College participants were recruited from local clinics, the laboratory website,^1^ and online parents’ forums. In Belgium, children were recruited from university hospital centers dedicated to treatment of children with CP. The parents/legal tutors of potential participants were contacted by e-mail or telephone. All parents/legal tutor and children provided their written, informed consent to participate in the study, which had been approved by the respective Institutional Review Boards.

Participants (see Table 1) were classified following the Manual Ability Classification System (Eliasson et al., 2006) as levels I (*n* = 8), II (*n* = 30) or III (*n* = 2). Children were also evaluated by a pediatric neurologist and a neuroradiologist using the criteria of Krageloh-Mann and Horber (2007), to classify the origin of their brain lesion (cortical malformation, *n* = 6; periventricular lesion, *n* = 18; cortical/subcortical lesion, *n* = 16).

**TABLE 1:** Group’s characteristics.

Lesion type	Gender	Age	MACS	Lesion side
Cortical/subcortical lesion (*n* = 16)	Girls = 9 Boys = 7	8 y 6 m	I = 5 II = 11	Right = 6 Left = 10
Periventricular lesion (*n* = 15)	Girls = 6 Boys = 9	9 y 6 m	I = 3 II = 12	Right = 8 Left = 7

**Lesion side**	**Gender**	**Age**	**MACS**	**Lesion type**

Right hemisphere (*n* = 16)	Girls = 9 Boys = 7	8 y 6 m	I = 5 II = 11	C/Sc = 6 PVL = 10
Left hemisphere (*n* = 19)	Girls = 9 Boys = 7	8 y 6 m	I = 5 II = 11	C/Sc = 6 PVL = 10

The inclusion criteria were (1) children diagnosed with unilateral CP, (2) aged 6–16 years, (3) ability to grasp light objects and lift the more affected arm 15 cm above a table surface, (4) school level equal to that of typically developing peers, (5) ability to follow instructions and complete testing. Exclusion criteria were (1) uncontrollable seizures, (2) botulinum toxin injections in the previous 6 months or planned for the following 6 months, (3) orthopedic surgery in the previous 12 months or planned within the study period, (4) uncorrectable visual problems likely to interfere with treatment/testing.

### 3D-magnetic resonance imaging and diffusion tensor imaging

In a single scanning session, children underwent an MRI scan at 3T with a 32-channel phased array head coil to record 3D heavily T1-weighted structural and diffusion tensor imaging (DTI) images. In Brussels, 26 children were scanned using a Philips Achieva magnet (Philips Healthcare, Eindhoven, The Netherlands). Nine children were scanned using a Philips Achieva at Columbia Medical School and five using a Siemens Prisma 3T scanner (Siemens, Erlangen, Germany) at Cornell-Weil Hospital in New York.

The anatomical 3D sequence obtained on the Philips magnet consisted of a gradient echo sequence with an inversion prepulse (Turbo Field Echo, TFE) acquired in the sagittal plane employing the following parameters: repetition time (TR) = 9.1 ms, echo time (TE) = 4.6 ms, flip angle = 8°, 150 slices, slice thickness = 1 mm, in-plane resolution = 0.81 × 0.95 mm^2^ (acquisition) reconstructed in 0.75 × 0.75 mm^2^, field of view (FOV) = 220 × 197 mm^2^, acquisition matrix = 296 × 247 (reconstruction 3202), SENSE factor = 1.5 (parallel imaging), with a total scan time of 8 min and 26 s. The anatomical 3D sequence obtained on the Siemens magnet consisted of a magnetization-prepared rapid gradient-echo (MP-RAGE) sequence acquired in the axial plane employing the following parameters: TR = 2170 ms, TE = 4.33 ms, flip angle = 7°, 176 slices, slice thickness = 1 mm, in-plane resolution = 1 × 1 mm^2^ (acquisition and reconstruction), FOV = 256 × 256 mm^2^, acquisition matrix = 256 × 256, GRAPPA factor = 2 (parallel imaging), in a 4 min 27 s of total scan time.

DTI images on the Achieva scanner were obtained using the following sequence: spin-echo planar imaging, TE = 83 ms, TR = 6,422 ms, Bandwidth = 2,790 hz/pixel, 70 slices, slice thickness = 2 mm, in-plane resolution = 2 × 2 mm^2^, matrix size = 112 × 112, FOV = 224 × 224 mm^2^, 55 directions, b = 800 s/mm^2^, in a total scan time of 8 min. DTI images on the Prisma scanner were acquired employing the following sequence: spin-echo planar imaging, TE = 83 ms, TR = 9,000 ms, Bandwidth = 1,860 hz/pixel, 75 slices, slice thickness = 2 mm, in-plane resolution = 2 × 2 mm^2^, matrix size = 112 × 112, FOV = 224 × 224 mm^2^, 64 directions, b = 1,000 s/mm^2^, in a total scan time of 10 min and 14 s.

The DTI data were pre-processed using BrainVoyager (Version 20.6, Brain Innovation, Maastricht, The Netherlands). The diffusion data were first corrected for eddy current-induced distortions and motion-induced artifacts. To create the fractional anisotropy (FA) map and to calculate the radial (RD), axial (AD), and mean diffusivity (MD), the DTI data were co-registered with the 3D anatomy of the subject without normalization (native space).

**FIGURE 1:**
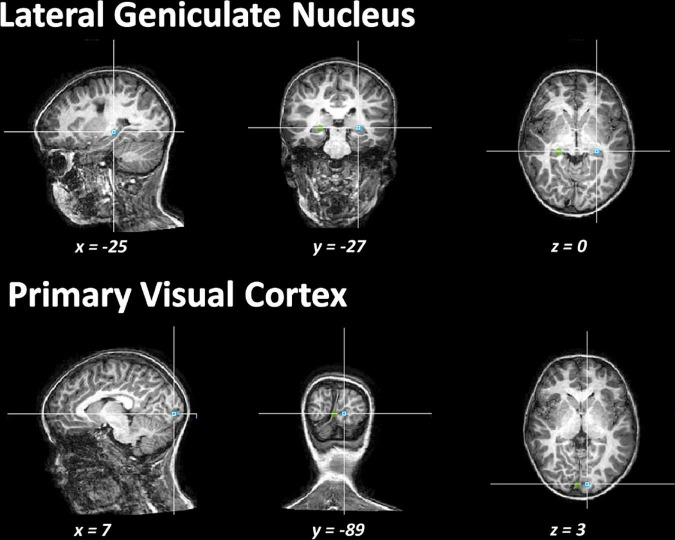
Illustration of the regions of interest drawn to execute the tracking of the fibers.

To determine individual measures of the DTI metrics (FA, MD, AD, and RD) of the OR within the lesional and non-lesional hemispheres, axial planes were first imposed based on anatomical landmarks (see Figure 1). For each hemisphere, we used two regions of interest (ROIs) to trace the OR: an initial ROI was defined at the level of the lateral geniculate nucleus and a second in the visual primary cortex near the calcarine sulcus. These regions were identified on the echo planar images with no diffusion weighting and were verified later, in all planes, by an investigator uninvolved in the tracking. Fibers passing over the lateral geniculate nucleus were excluded from the analysis. A deterministic tracking was finally performed to reconstruct and track the OR. Only those fibers with FA > 0.20 and a deviation angle < 50° were included in the tracking. The MRI acquisition was performed by examiners unaware of the study purpose.

### Visuospatial assessment

We used a star cancelation test to assess impairments in visuospatial attention specifically related to unilateral spatial neglect. In this test, the child is asked to cancel all small stars in a sheet of paper marked with stars of two different sizes. This test separately records the total number of omissions as well as the omissions on the more affected and the less affected hemispaces (Wilson et al., 1987).

### Data analysis

#### Diffusion tensor imaging analyses

First, a *t*-test (or Mann-Whitney rank sum test when the normality was not respected) was performed to identify possible differences between the OR scores of the lesional hemisphere (OR-LH) and the non-lesional hemisphere (OR-NLH) for FA, MD, AD, and RD. Next, we used a two-way ANOVA to identify the influence of type of lesion (periventricular/cortical-subcortical) on the differences between the OR-LH and the OR-NLH scores for FA, MD, AD, and RD. We performed Student-Newman-Keuls *post hoc* tests, after adjustment of the alpha level for multiple comparisons. Finally, we performed a two-way ANOVA with a Student-Newman-Keuls *post hoc* (α = 0.05) to identify the influence of side of lesion (right/left) in the differences between the OR-LH and the OR-NLH scores for FA, MD, AD, and RD.

#### Visuospatial assessment analyses

We used a Mann-Whitney rank sum test to evaluate impairment in visuospatial attention in relation to unilateral spatial neglect. Here, we separately compared each result of the star cancelation test (total number of omissions, more affected hemispace omission and less affected hemispace omission) with lesion type (periventricular or cortical/subcortical lesion) and lesion side (right/left).

#### Correlation analyses

A Spearman’s correlation was performed to investigate the relationships between the visuospatial test results and DTI measures (FA, MD AD, and RD) in the OR-LH and in the OR-NLH as functions of the lesion type and side.

## Results

After the data acquisition, five children were excluded from the analyses because of inadequate quality of neuroimaging data produced mainly by head movement. Therefore, the final sample for the analyses was of 35 children.

### Description of optic radiations organization

**FIGURE 2:**
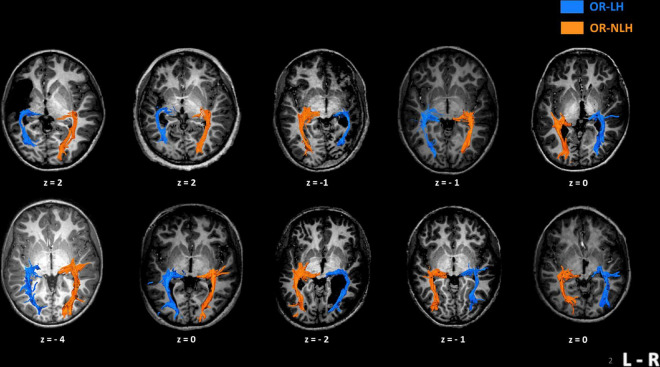
Imaging results in 10 children with unilateral spastic cerebral palsy, displaying their individual optic radiation bypasses. The upper row shows examples of children with cortico-subcortical lesions and the lower row depicts children with periventricular lesions. *Z*-values represent the coordinates in Talairach space. OR-LH, optic radiation of the lesional hemisphere; OR-NLH, optic radiations of the non-lesional hemisphere; L-R, left-right.

For every participant, we could identify the OR between the lateral geniculate nucleus and the visual cortex, regardless of the type and side of lesion. We distinguished two types of lesion “bypasses” to the visual cortex; in children with a cortico-subcortical lesion, the OR-LH passed through the lesion, while in children with a periventricular lesion, the OR-LH systematically followed the outer contour of the lesion (Figure 2).

### Interhemispheric differences in diffusion tensor imaging measures

**FIGURE 3:**
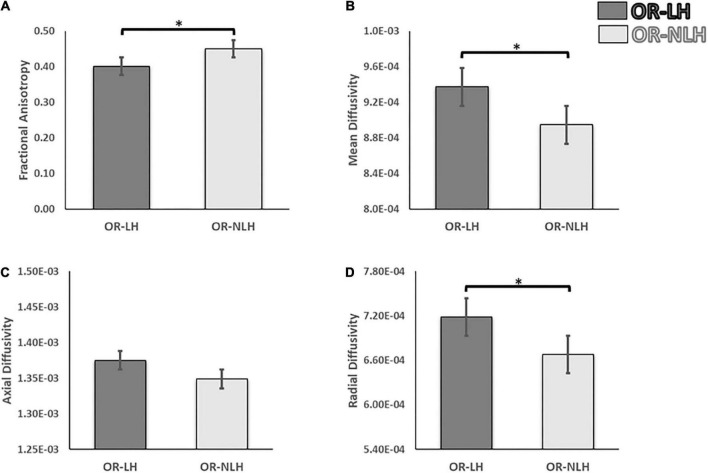
Differences in white matter integrity of the OR of the lesional and non-lesional hemispheres in children with USCP. **(A)** Mean fractional anisotropy, **(B)** mean diffusivity **(C)** mean axial diffusivity and **(D)** mean radial diffusivity. OR-LH, optic radiation of the lesional hemisphere; OR-NLH, optic radiations of the non-lesional hemisphere. Error bars represent standard errors of the mean. *p < 0.05.

We observed significant differences in DTI parameters of the OR-LH and OR-NLH. The FA values were lower in OR-LH compared to OR-NLH (Mann-Whitney U = 261.00, *p* < 0.001). Conversely, there were higher values in OR-LH compared to OR-NLH for MD (Mann-Whitney U = 433.00, *p* < 0.05) and RD (Mann-Whitney U = 424.00, *p* < 0.05). No significant difference were observed for the AD (Mann-Whitney U = 549.00, *p* = 0.459) (Figure 3).

#### Influence of lesion type

The final sample implicated in the analyses of the influence of the lesion was of 31 children; due to the small sample, children presenting a brain malformation were excluded from this analysis.

**FIGURE 4:**
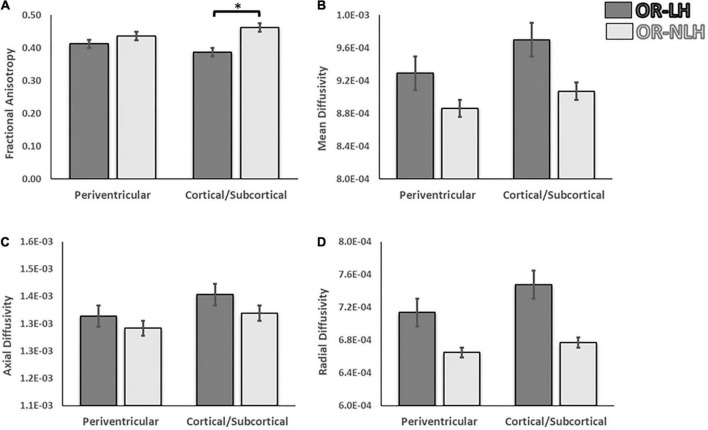
Differences in white matter integrity of the OR of the lesional and non-lesional hemispheres as functions of lesion type in children with USCP. **(A)** Fractional anisotropy, **(B)** mean diffusivity, **(C)** mean axial diffusivity and **(D)** mean radial diffusivity. OR-LH, optic radiation of the lesional hemisphere; OR-NLH, optic radiations of the non-lesional hemisphere. Error bars represent standard errors of the mean. *p < 0.05.

To identify the influence of the type of lesion on the differences in the FA, MD, AD, and RD between the OR-LH and the OR-NLH, we performed a 2 (optic radiations condition: OR-LH vs. OR-NLH) × 2 (type of lesion: periventricular vs. cortical/subcortical) ANOVA. These analyses revealed an optic radiations condition effect on FA values [*F*(1, 58) = 18.30; *p* < 0.001, η^2^ = 0.102]. Lesion type had no such effect [*F*(1, 58) = 0.01; *p* = 0.993, η^2^ = 0.131], but there was a significant optic radiations condition × lesion type interaction effect [*F*(1, 58) = 4.63; *p* < 0.05, η^2^ = 0.174]. Figure 4 shows the results of the *post hoc* analysis. In cases of cortico-subcortical lesions (*p* < 0.001) mean FA values were lower in the OR-LH compared to OR-NLH.

The same analyses for the diffusivity showed an effect on the MD for the optic radiations condition [*F*(1, 58) = 5.17; *p* < 0.05, η^2^ = 0.152] with lower values for the OR-NLH, but no significant difference for the lesion type [*F*(1, 58) = 1.77; *p* = 0.19, η^2^ = 0.130] or interaction [*F*(1, 58) = 0.18; *p* = 0.671, η^2^ = 0.103]. In addition, for the AD a trend was observed for the optic radiations condition [*F*(1, 58) = 3.55; *p* = 0.65, η^2^ = 0.155], but a significant effect on the lesion type conditions [*F*(1, 58) = 5.00; *p* < 0.05, η^2^ = 0.229] with higher values for the cortical/subcortical lesion and no interaction [*F*(1, 58) = 0.15; *p* = 0.701, η^2^ = 0.121]. Also, a significant effect was observed for the RD for the optic radiations conditions [*F*(1, 58) = 4.92; *p* < 0.05, η^2^ = 0.178], but none for the lesion type conditions [*F*(1, 58) = 0.74; *p* = 0.394, η^2^ = 0.113] and no interactions [*F*(1, 58) = 0.16; *p* = 0.688, η^2^ = 0.116] (Figure 4).

#### Influence of lesion side

**FIGURE 5:**
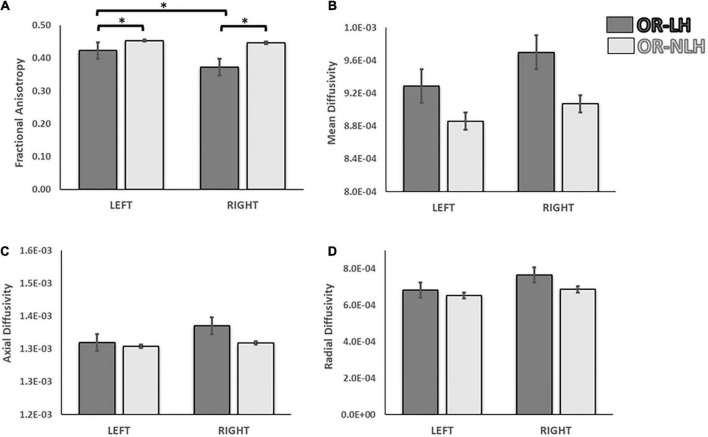
Differences in white matter integrity of the OR of the lesional and non-lesional hemispheres as functions of side of lesion in children with USCP. **(A)** Fractional anisotropy, **(B)** mean diffusivity, **(C)** mean axial diffusivity and **(D)** mean radial diffusivity. OR-LH: optic radiation of the lesional hemisphere; OR-NLH: optic radiations of the non-lesional hemisphere. Error bars represent standard errors of the mean. *p < 0.05.

To test for effects of lesion side on group differences between the OR-LH and the OR-NLH group means of the FA, MD, AD, and RD, we performed a 2 (optic radiations condition: OR-LH vs. OR-NLH) × 2 (side of lesion: right vs. left) ANOVA. We found a significant effect on the FA of the OR condition [*F*(1, 66) = 24.98; *p* < 0.001, η^2^ = 0.275], lesion side conditions [*F*(1, 66) = 7.64; *p* < 0.05, η^2^ = 0.204] and a significant interaction effect between the OR condition and side of the lesion [*F*(1, 66) = 4.37; *p* < 0.05, η^2^ = 0.192]. As depicted in Figure 5, children with a right lesion had lower values in OR-LH (*p* < 0.001) compared with children with a left lesion. Additionally, we observed lower mean FA values in OR-LH compared with the OR-NLH in children with a right (*p* < 0.001) and a left (*p* < 0.05) lesion.

The same analyses for the diffusivity showed an effect on the MD for the optic radiations condition [*F*(1, 66) = 4.62; *p* < 0.05, η^2^ = 0.265] with lower values for the OR-NLH and significant difference for the lesion side [*F*(1, 66) = 5.10; *p* < 0.05, η^2^ = 0.272] with lower values for the left lesion, but no interaction [*F*(1, 66) = 1.20; *p* = 0.278, η^2^ = 0.119]. There were no significant effects of OR condition [*F*(1, 66) = 2.07; *p* = 0.155, η^2^ = 0.130], lesion side [*F*(1, 66) = 1.95; *p* = 0.167, η^2^ = 0.127] or their interaction [*F*(1, 66) = 1.08; *p* = 0.302, η^2^ = 0.116] for AD results. In addition, for the RD a significant effect was observed for the optic radiations condition [*F*(1, 66) = 5.04; *p* < 0.05, η^2^ = 0.271] with lower values for the OR-NLH and a significant effect on the lesion type conditions [*F*(1, 66) = 5.79; *p* < 0.05, η^2^ = 0.282] with lower values for the left lesion and no interaction [*F*(1, 58) = 0.15; *p* = 0.701, η^2^ = 0.115].

### Visuospatial assessment

Children with USCP showed a mean of 2.91 ± 3.64 total omissions in the star cancelation test. These results are in line with those described in the literature in children with USCP (Ickx et al., 2018). Compared to aged-matched typically developing peers, only few children presented abnormal values (Ickx et al., 2017). Lesion side and type had no effect on the total number of omitted small stars, neither in the more affected nor the less affected hemispace (all *p* > 0.235, Table 2; see also Table 3).

**TABLE 2:** Star cancelation scores.

All children	Total omissions		
(*n* = 35)	mean (SD)		
	2.91 (3.64)		

**Lesion side**	**Total omissions**	**More affected hemispace omissions**	**Less affected hemispace omissions**
	Median [25–75%]	Median [25–75%]	Median [25–75%]
Right hemisphere (*n* = 16)	2.00 [0.00–4.00]	1.00 [0.00–4.00]	0.00 [0.00–1.00]
Left hemisphere (*n* = 19)	1.50 [0.00–5.00]	0.50 [0.00–2.00]	0.50 [0.00–2.00]
*p-value*	0.904	0.412	0.235

**Lesion type**	**Total omissions**	**More affected hemispace omissions**	**Less affected hemispace omissions**

	Median [25–75%]	Median [25–75%]	Median [25–75%]
Cortical/subcortical lesion (*n* = 16)	2.50 [0.00–5.00]	1.00 [0.00–4.00]	0.00 [0.00–2.50]
Periventricular lesion (*n* = 15)	1.00 [0.00–5.00]	1.00 [0.00–2.00]	0.00 [0.00–3.00]
*p-value*	0.822	0.563	0.912

**TABLE 3:** Amount of children presenting impaired visuospatial assessment.

Lesion type	Total omissions	More affected hemispace omissions	Less affected hemispace omissions
Cortical/subcortical lesion (*n* = 16)	3	3	2
Periventricular lesion (*n* = 15)	4	4	1

**Lesion side**	**Total omissions**	**More affected hemispace omissions**	**Less affected hemispace omissions**

Right hemisphere (*n* = 16)	3	2	0
Left hemisphere (*n* = 19)	4	4	3

### Correlation between diffusion tensor imaging measures and visuospatial assessment

In children with a periventricular lesion, the FA of the OR-LH correlated significantly with the total number of stars omitted (*r* = –0.546; *p* = 0.034) and the FA of the OR-NLH correlated significantly with the total number of stars omitted (*r* = –0.638; *p* = 0.01).

In children with a right-side brain lesion, the MD of the OR-NLH correlated significantly with the total number of stars omitted (*r* = 0.536; *p* = 0.038) and the RD of the OR-NLH with the total number of stars omitted (*r* = 0.561; *p* = 0.029). There were no other significant correlations with test scores.

## Discussion

The aim of this study was to assess the organization and white matter characteristics of the OR in children with USCP, and test for an association with impairment in their performance of a visuospatial test. We also tested the hypothesis that type and side of the lesion would influence parameters of white matter microstructure, i.e., FA, MD, AD, and RD. Our results support this hypothesis, highlighting differences in the white matter properties of the OR between the lesional and the non-lesional hemisphere. These microstructural differences were influenced by the type of lesion and the side of the lesion, and may contribute to the visuospatial impairments often observed in children with USCP.

**FIGURE 6:**
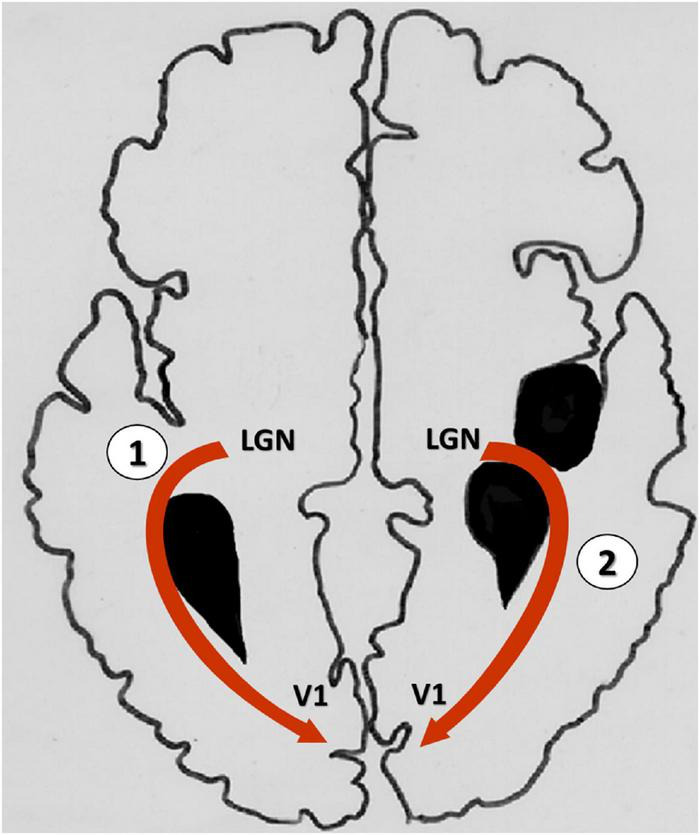
Graphical representation of the two bypass routes of the optic radiations in the lesional hemisphere. **(1)** Optic radiations contouring the edge of lesion to reach the primary visual cortex; **(2)** Optic radiations passing through the lesion attain the visual cortex. LGN, Lateral geniculate nucleus; V1, primary visual cortex.

We observed two different patterns of white matter reorganization, apparently in response to the lesion. In one scenario, OR fibers in the lesion hemisphere passed through cortico-subcortical injuries, and in other cases, the OR followed the contour periventricular lesions (Figure 6). This last finding is in agreement with the proposal by Guzzetta (2010) that, when the lesion affects the retro-geniculate pathway, the OR circumvents the lesion contours to reach the primary visual cortex (Guzzetta, 2010). Moreover, in our study the two bypass routes also differed with respect to the microstructure of white matter fibers, as indicated by FA values. We suggest that the distinct modes of reorganization of the visual pathway may bear relation to the timing and the location of the lesion (Chugani et al., 1996; Feys et al., 2010). Indeed, an association between reorganization and lesional features is critical for the development of motor, sensory as well as cognitive deficits in children with USCP (Lidzba et al., 2006; Guzzetta et al., 2007; Staudt, 2010; Hadders-Algra, 2014). Thus, the importance of lesion features for reorganization of the visual pathway is a novel finding of this study, demonstrating the far-reaching consequences of early brain lesions in children with CP.

In this study we observed a significant difference between the OR-LH and OR-NLH, with the OR-LH showing lower values in the FA and higher values in MD and RD, but no difference in AD. These results indicate an impact of the brain lesion on the structure of the OR. The FA difference could indicate an impairment of the white matter microstructure, although this measure is affected by many factors including myelination, axon size, and density (Beaulieu, 2002; Mukherjee et al., 2002; Ciccarelli et al., 2008). However, the results observed in the FA were accompanied with higher values in the MD and RD of OR-LH without significant differences in AD. The RD has been associated with the myelin (Song et al., 2002, 2005) and AD with axonal microstructure (Sun et al., 2008; Budde et al., 2009). Therefore, our results suggest that the low FA associated with higher values in the AD and MD observed in OR-LH are potentially manifestations of impaired microstructure associated with altered myelin development process.

In this study, we observed lower FA values in OR-LH of children with a cortico-subcortical lesions compared to those with a periventricular lesion. The differences in FA between the lesional and non-lesional hemispheres is in line with previous studies showing an association between early brain lesions and damage to OR, specifically in children with lesions in the periventricular area (Leviton and Paneth, 1990; Rodriguez et al., 1990; Cioni et al., 2000). Our results are also consistent with findings of Chokron and Dutton (2016), indicating a correlation between white matter lesions in OR and impaired visual function in children with CP.

The difference in FA could relate to the specific cortical phase of development at the time of brain damage, i.e., start of 3rd trimester for periventricular lesions and end of 3rd trimester for cortical/subcortical lesions. Myelinization of the OR begins around the middle of the 3rd trimester (de Graaf-Peters and Hadders-Algra, 2006), such that a lesion occurring at around the 35th week of gestation is apt to have a higher impact on white matter in childhood. Similar differences between these two broad types of lesion have been shown in relation to motor function, since impairments of upper extremity function (Feys et al., 2010) and language skills (Coleman et al., 2013) are more common in CP children with cortical/subcortical compared to periventricular lesions. It is noteworthy that the myelination of projections from the precentral and postcentral gyrus, which are key substrates of sensorimotor functions, also begins during the 35th gestational week (de Graaf-Peters and Hadders-Algra, 2006). Damage occurring after the onset of myelination thus likely results in larger deficits than do earlier injuries. This phenomenon may represent a general pattern whereby lesions occurring prior to onset of the myelination process may have less impact on white matter and on functional abilities, while lesions occurring after the start of myelination are more deleterious. The difference observed between both lesion types could be related with the injury consequences such as microglial activation, excitotoxicity and free radical that produced, among other effects, damage of the oligodendrocyte and/or its precursors that play a crucial role in the myelination (Volpe et al., 2011). However, the consequences of the different lesion types for oligodendrocyte development are not completely understood (Silbereis et al., 2010).

Although we saw systematically larger deficits in the OR of the lesional vs. the non-lesional hemisphere, deficits in the OR of the lesional hemisphere were larger in children with right hemispheric lesions. These results indicate an impact of the lesion side on the characteristics of the OR fibers. A larger impact of right hemispheric lesions on visuospatial skills has been described in children with USCP (Kolk and Talvik, 2000), suggesting that a lesion of the right hemisphere could drive changes in the OR or other white matter structures involved in visuospatial abilities (Tuch et al., 2005). However, children with USCP with a left hemispheric lesion also show deficits in visuospatial abilities (Thareja et al., 2012). This could be explained by the “crowding hypothesis,” according to which a lesion in the left hemisphere likely produces a functional shift of the areas normally subserving language from the left to the right hemisphere, which may compromise visuospatial function of the right hemisphere (Lidzba et al., 2006). Therefore, a lesion of either hemisphere could impact the OR and thus affect visuospatial abilities, but by different mechanisms. This sensitivity of visuospatial function to lesion in either hemisphere is predictable from the requirement of intra-hemispheric integration for a bilateral representation of visual space (Corbetta and Shulman, 2011). In this scenario, the lesional side could influence the FA or other aspects of OR microstructure in children with USCP. We suggest that, although lesions on either hemisphere may have an impact on OR microstructure, the effects of right hemispheric lesions are greater due to the specialization of the right hemisphere for visuospatial function. However, despite the different characteristics of the lesion are influenced by the stage of brain maturation, the effects of the lesion may be influenced by other characteristics of the lesion such as the location, size and mechanisms involved (Graham et al., 2016).

At a behavioral level, different studies have shown a deficit in children with cerebral palsy on visuospatial assessments compared with reference values or control peers (Lidzba et al., 2006; Ickx et al., 2017, 2018). In addition, in this study we observed some correlation between FA, MD and RD with the number of omissions in the star cancelation test, suggesting a possible association between visuospatial attention deficits, specifically related to visuospatial impairments, and the damage of the white matter projections of the visual pathway. Tuch et al. (2005) showed a correlation between reaction times in a visual task and FA of the OR in healthy adult subjects, demonstrating that the OR mediates aspects of visual attention (Tuch et al., 2005). This is congruent with other studies of children with CP showing cortical visual impairments due to damage to the retrochiasmatic part of the visual pathway (Mercuri et al., 1996; Guzzetta et al., 2001), particularly in the OR (Ramenghi et al., 2010). However, the development of visual functions, including visuospatial attention, requires the integrity of a wide cortical and subcortical network, including the OR and the primary visual cortex, but also involving frontal and temporal regions, as well as the basal ganglia (Ramenghi et al., 2010). Therefore, while the present finding of disturbances in the OR of children with USCP are likely relevant to their visuospatial impairments, the OR is but one element among many.

Finally, this study presents some limitations regarding the information about the cerebral palsy cause and the extension of the lesion that could contribute to better understand the development of visuospatial functions. In addition, the assessment used in this study could have been complemented with a visual fields assessment, as well as, we could have used more than one test to evaluate the deficit in visuospatial attention. However, the knowledge about the visuospatial function should contribute to open a new perspective to understand the relevance of this function in rehabilitation programs.

## Conclusion

We found differences in white matter characteristics of the OR between the lesional and the non-lesional hemispheres of children with USCP. These differences were apparently influenced by the timing of the lesion and by the side of the lesion, perhaps due to competition between language and visuospatial function. The observed differences may contribute to the different degrees of visuospatial impairments observed in children with USCP. We suggest that the timing of the lesion relative to myelination landmarks contributes importantly to the outcome for these children, probably in relation to compensation for injury to visual pathways through neuroplastic changes.

## Data availability statement

The data will be made available upon publication to researchers who provide a methodologically sound proposal for use in achieving the goals of the approved proposal. Proposals should be submitted to YB, yannick.bleyenheuft@uclouvain.be.

## Ethics statement

The studies involving human participants were reviewed and approved by the Comité d’Ethique Hospitalo-Facultaire, Université Catholique de Louvain, Brussels, Belgium and Teachers College, Columbia University Institutional Review Board, New York, United States. Written informed consent to participate in this study was provided by the participants’ legal guardian/next of kin.

## Author contributions

RA and DE-K participated to data collection, data analysis, and writing. LD participated to project design, data collection, data analysis, and writing. EH participated to data collection and writing. SMH, KMF, and AMG participated to project design and writing. YB participated to project design, recruitment, data collection, data analysis, and writing. All authors contributed to the article and approved the submitted version.

## Footnotes

1
www.tc.edu/centers/cit/

## Abbreviations

USCP: unilateral spastic cerebral palsy
OR: optic radiation
FA: fractional anisotropy
MD: mean diffusivity
CP: Cerebral palsy
ROI: regions of interest
OR-LH: optic radiation scores on the lesional hemisphere
OR-NLH: optic radiation scores on the non-lesional hemisphere.
